# Electrosome assembly: Structural insights from high voltage-activated calcium channel (Ca_V_)–chaperone interactions

**DOI:** 10.1042/BST20240422

**Published:** 2025-02-06

**Authors:** Zhou Chen, Daniel L. Minor,

**Affiliations:** 1 Cardiovascular Research Institute, University of California-San Francisco, San Francisco, CA 94158-9001, U.S.A; 2 Department of Biochemistry and Biophysics, and Cellular and Molecular Pharmacology, University of California-San Francisco, San Francisco, CA 94158-9001, U.S.A; 3 California Institute for Quantitative Biomedical Research, University of California-San Francisco, San Francisco, CA 94158-9001, U.S.A; 4 Kavli Institute for Fundamental Neuroscience, University of California-San Francisco, San Francisco, CA 94158-9001, U.S.A; 5 Molecular Biophysics and Integrated Bio-imaging Division Lawrence Berkeley National Laboratory, Berkeley, CA 94720 CA 94720, U.S.A

**Keywords:** EMC, ion channel assembly, molecular chaperones, voltage-gated ion channel

## Abstract

Ion channels are multicomponent complexes (termed here as“electrosomes”) that conduct the bioelectrical signals required for life. It has been appreciated for decades that assembly is critical for proper channel function, but knowledge of the factors that undergird this important process has been lacking. Although there are now exemplar structures of representatives of most major ion channel classes, there has been no direct structural information to inform how these complicated, multipart complexes are put together or whether they interact with chaperone proteins that aid in their assembly. Recent structural characterization of a complex of the endoplasmic membrane protein complex (EMC) chaperone and a voltage-gated calcium channel (Ca_V_) assembly intermediate comprising the pore-forming Ca_V_α_1_ and cytoplasmic Ca_V_β subunits offers the first structural view into the assembly of a member of the largest ion channel class, the voltage-gated ion channel (VGIC) superfamily. The structure shows how the EMC remodels the Ca_V_α_1_/Ca_V_β complex through a set of rigid body movements for handoff to the extracellular Ca_V_α_2_δ subunit to complete channel assembly in a process that involves intersubunit coordination of a divalent cation and ordering of Ca_V_α_1_ elements. These findings set a new framework for deciphering the structural underpinnings of ion channel biogenesis that has implications for understanding channel function, how drugs and disease mutations act, and for investigating how other membrane proteins may engage the ubiquitous EMC chaperone.

## Introduction

**Figure 1:**
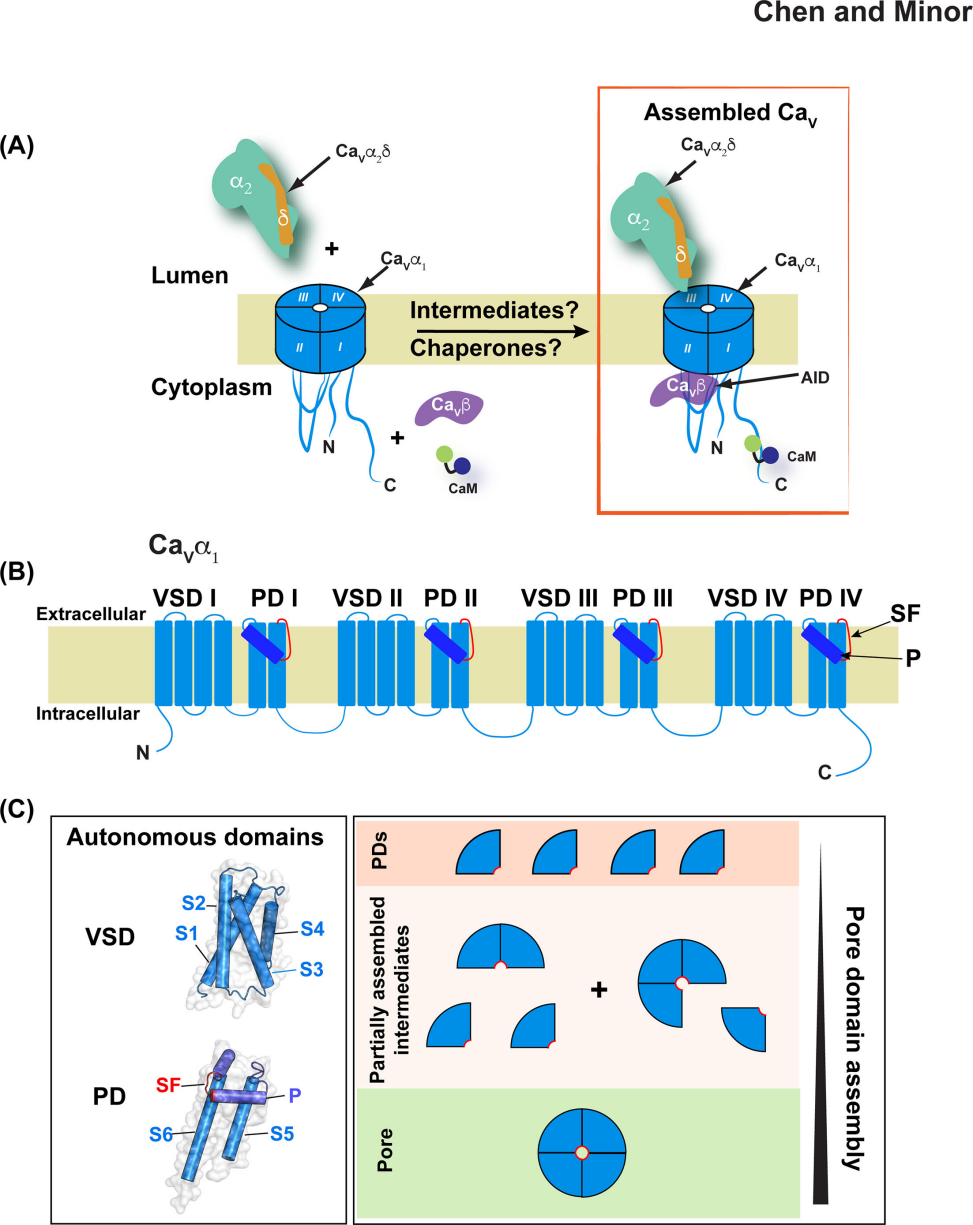
Voltage-gated ion channel assembly. **(A)** Ca_V_ subunit assembly. (Left) Cartoon of the Ca_V_1s and Ca_V_2s subunits: pore-forming Ca_V_α_1_, extracellular Ca_V_α_2_δ, and intracellular Ca_V_β subunits and calmodulin (CaM) are shown. (Right), Assembled Ca_V_. High affinity Ca_V_β binding site, α-interaction domain (AID) is indicated. **(B)** Ca_V_α_1_ pore-forming subunit schematic. Voltage sensor (VSD) and pore domain (PD) elements are indicated. PD pore helix (**P**) (dark blue) and Selectivity filter (SF) (red) are indicated. **(C)** (left) Exemplar autonomously folded VGIC domains: VSD (PDB:4G80) [4] and PD (PDB:7PGB) [5]. (right) Progression of intermediates in VGIC pore assembly. SF element is indicated in red.

Ion channels are multisubunit protein complexes that create the spark of life by producing the electrical signals that drive our thoughts, feelings, and actions. Thanks to more than two decades of effort, there are now representative structures for most of the major ion channel classes that provide a framework for functional studies and modulator development. Nevertheless, how these multi-component signaling molecules, which we term “electrosomes”, are put together and undergo quality control to ensure that all of the correct parts are assembled remains largely unaddressed [1]. Among the ion channel types, voltage-gated ion channels (VGICs) form the largest ion channel superfamily [2] encompassing various classes of voltage-gated calcium (Ca_V_s), sodium (Na_V_s), and potassium (K_V_s) channels, the TRP and TPC channel families, inwardly rectifying potassium channels, and K_2P_ leak potassium channels. All VGIC superfamily members share a common four-fold, barrel stave body plan in which four pore domain (PD) subunits each bearing part of the selectivity filter (SF) assemble around the central axis of the channel, making the hole through which the ions pass. The four PDs can come from four individual polypeptides (K_V_s, Kirs, TRPs), or from polypeptides that carry two (K_2P_s and TPCs) or all four PDs (Ca_V_s and Na_V_s). In addition to the PD, many channel classes have voltage sensor domains (VSDs) or voltage sensor-like domains fused to each PD. Adding further complexity, numerous channel types, such as high-voltage-activated Ca_V_1 and Ca_V_2s, also have auxiliary subunits that are required to make the final, functioning multiprotein complex [3] (Figure 1A). This multitude of parts required to form a VGIC superfamily channel means that during the initial phase of its life, there must be some set of partially assembled intermediates. Until recently, there was no structural information about any ion channel assembly intermediate, or clear indication about the extent to which chaperone proteins might play a role in channel assembly. Here, we briefly outline key structural issues regarding ion channel biogenesis and focus on studies uncovering the interaction of a class of paradigmatic electrosomes, Ca_V_s, with a ubiquitous endoplasmic reticulum (ER) chaperone known as the endoplasmic reticulum membrane protein complex (EMC) that provides the first structural insight into the ion channel assembly process.

### Fundamental issues for ion channel assembly

To make a channel, the first thing that must happen is that the pore-forming subunit has to be synthesized by the ribosome and inserted into the ER membrane [6]. The average speed of eukaryotic protein synthesis of ~5.6 amino acids/sec [7,8] sets a biogenic “speed limit” for how quickly a protein of interest can be made that has direct consequences for the folding and assembly of multicomponent proteins such as ion channels. It takes ~3 minutes to make a thousand amino acid protein (for reference, many Ca_V_ and Na_V_s pore-forming subunits are ~2000–3000 amino acids long) [5]. Regardless of whether all the pore-forming components are in one or multiple polypeptide chains, the fact that the protein is made in a linear fashion means that there must be some period during the synthesis when the various VSDs and PDs are incorporated into the membrane but lack their partners for final assembly. For example, for a channel such as a Ca_V_ or Na_V_ in which all four VSDs and PDs are contained in one polypeptide, the time between completing the synthesis of the first and fourth PDs is at least 3.4 minutes [5], leaving the first few PDs without their complete complement of partners for some interval. What happens with these components during this time, particularly the PDs where four are required to make the final pore structure? Can VSDs and PDs fold before assembling into the final quaternary structure? Are partially assembled states protected by chaperone proteins from making aberrant assemblies? How does the cell identify channel parts on the way to being assembled versus leftover pieces from incomplete assembly that need to be disposed? Although the importance of these questions has been appreciated [1,6], there has been scant experimental data to address most of these issues, leaving the ion channel biogenesis as one of the largest blind spots in our understanding of how these critical components of electrical signaling function and how disease mutations or drugs might affect this crucial part of the life of a channel.

### Ion channel assembly from pieces

High voltage-activated Ca_V_s (Ca_V_1 and Ca_V_2) [3] are ubiquitous elements of electrically excitable tissues such as muscle, brain, and heart and exemplify the idea of a multisubunit electrosome, comprising three subunits, the pore-forming Ca_V_α_1_, cytoplasmic Ca_V_β [9], and extracellular Ca_V_α_2_δ [10] subunits, and the calcium sensor calmodulin [11] (Figure 1A). It has been known for >30 years that Ca_V_β and Ca_V_α_2_δ association with Ca_V_α_1_ profoundly shapes Ca_V_ biophysical properties and plasma membrane expression [9,12–16]. The domain organization of the 24 transmembrane segment Ca_V_ pore-forming subunit (Figure 1B) into VSDs and PDs highlights the “channel by parts” architecture found throughout the VGIC superfamily in which distinct VSD and PD elements are readily identified [2]. Accordingly, structural data show that both VSDs [4,17–20] and PDs [5] are autonomously folded subdomains (Figure 1B) that are capable of adopting native or native-like tertiary structure in the absence of the quaternary interactions that make the final, folded channel. VSD folding autonomy is not surprising, as their four-helix bundle topology places their domain components near each other in the primary sequence making them self-contained subdomains. In contrast, the ion-conducing pore is only formed only when four PDs assemble, raising the possibility that PD folding could depend on the interdomain interactions formed in the final pore structure. Recent structural studies defining noncanonical quaternary assemblies of bacterial voltage-gated sodium channel (BacNa_V_) PDs [5] has shown that the two-transmembrane/P-helix PD architecture folds independently of the extensive quaternary interactions found in the final tetrameric pore (Figure 1B), in line with prior biochemical studies indicating that native-like topology can develop within a single PD subunit [21–23]. Hence, it appears that the initial assembly of the channel pore can proceed by an “assembly by parts” path in which autonomously folded VSDs and PDs come together following their synthesis to make the stereotypic quaternary structures found throughout the VGIC superfamily.

Because four PDs need to be assembled around the channel central axis to create the pore during channel biogenesis, there must be partially assembled pore intermediates comprising two and three PDs (Figure 1C). Such forms have not yet been observed structurally and determining how they form, how long lived they are, and whether they interact with chaperone proteins that could prevent spurious interactions with other membrane components remains an interesting and unaddressed question. Further, in cases like Ca_V_s where the pore is an obligate hetero-tetramer, whether there is a preferred order for how the PDs assemble is not known. Developing strategies to trap such intermediates to probe their properties is an important goal to understand how channel pores are put together and undergo quality control. Finally, for channels such as Ca_V_s that require subunits beyond the pore-forming one, the various parts need to be put together. In the case of Ca_V_s, this means forming a complex in which the intracellular Ca_V_β subunit [9] and extracellular Ca_V_α_2_δ subunit [10] are added to the pore-forming Ca_V_α_1_ (Figure 1A). Both interactions control the functional expression channel and understanding how such assembly happens offers not only insight into a fundamental step in the life of a channel but also may open opportunities to target such partially assembled states to control channel function. For this reason, the recent report of the first view of a Ca_V_ assembly intermediate [24] establishes a new direction for addressing the multifaceted means by which cells control their electrical excitability.

### Structure of a chaperone-bound ion channel assembly intermediate

**Figure 2:**
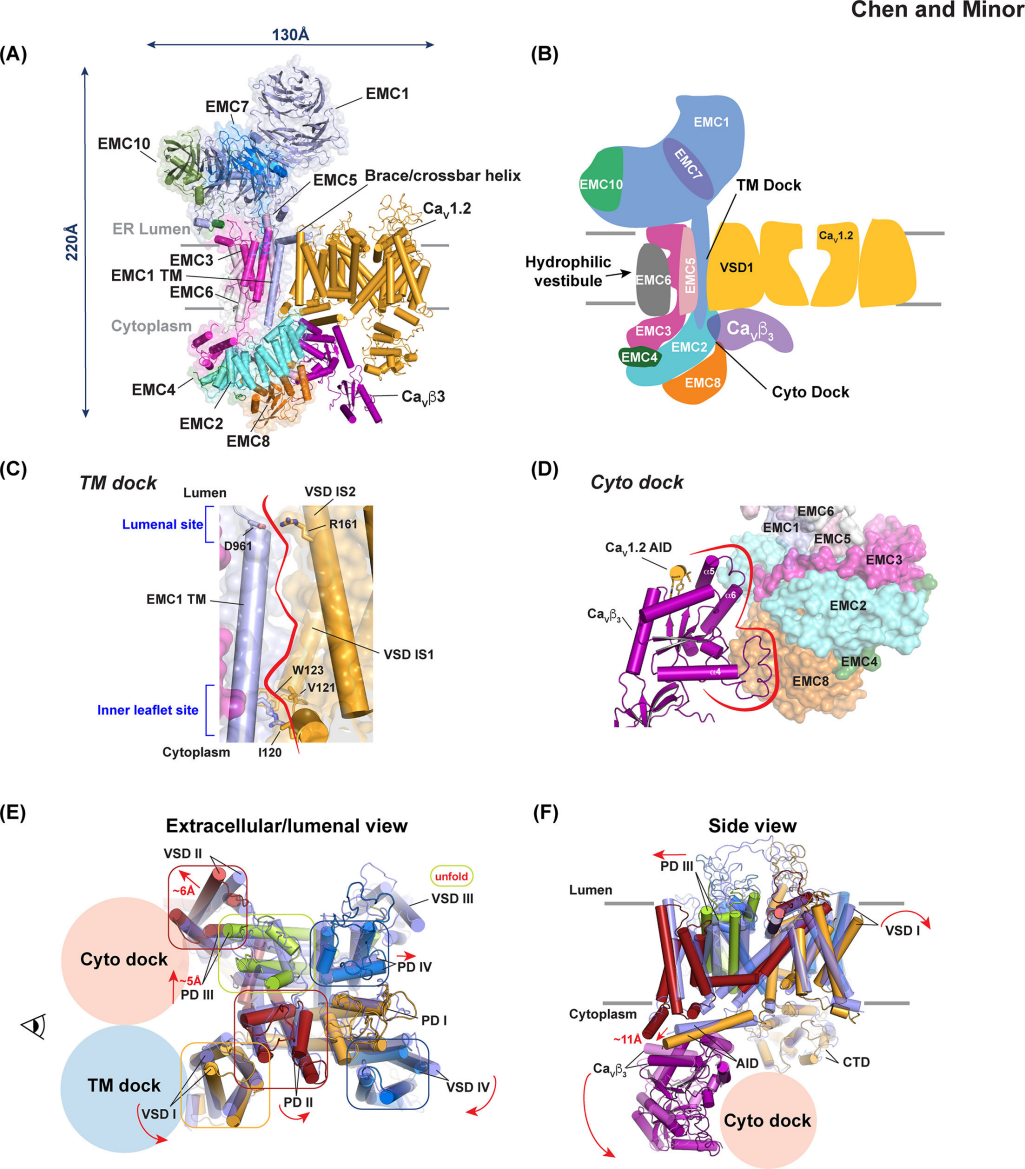
EMC:Ca_V_ complex—structure and conformational changes. **(A)** Structure of the EMC:Ca_V_1.2(ΔC)/Ca_V_β_3_ complex (PDB:8EOI) [24]. EMC1 (light blue), EMC2 (aquamarine), EMC3 (light magenta), EMC4 (forest green), EMC5 (light pink), EMC6 (white), EMC7 (marine), EMC8 (orange), EMC10 (smudge), Ca_V_1.2 (bright orange), and Ca_V_β_3_ (purple) are shown in cartoon rendering. EMC subunits are shown with semi-transparent surfaces. The arrows indicate dimensions. **(B)** Cartoon schematic of **(A)** showing TM dock and Cyto dock locations. Hydrophilic vestibule used for transmembrane segment insertion [36] is indicated and is on the opposite face of the EMC from the TM dock site. Colors as in **(A)**. **(C)** Side view of the TM dock–VSD I interface. EMC1 transmembrane (TM) and Ca_V_1.2 VSD helices are shown as cylinders. Lumenal and Inner leaflet site hydrophilic interactions are shown. Red line indicates the interface. **(D)** Cyto dock interface. Ca_V_β_3_ and AID helix are shown as cylinders. EMC1, EMC2, EMC3, EMC4, EMC5, EMC6, and EMC8 are shown as surfaces. Red line indicates the interface. **(E)** Superposition of Ca_V_1.2(ΔC)/Ca_V_β_3_ from the EMC complex (PDB:8EOI) [24] and assembled Ca_V_1.2 (PDB:8EOG) [24]. Ca_V_1.2 elements from the EMC complex are: VSD I/PD I (yellow orange), VSD II/PD II (dark red), VSD III/PD III (lime), and VSD IV/PD IV (deep blue), Ca_V_β_3_ (purple). Ca_V_1.2 (marine) and Ca_V_β_3_3 (magenta) from the Ca_V_1.2(ΔC)/C Ca_V_β_3_/Ca_V_α_2_δ-1 assembled channel are semi-transparent. Red arrows indicate conformational changes from the assembled channel to the EMC complex. Ovals highlight domains that undergo conformational changes. TM dock and Cyto dock positions are indicated by the blue and red-orange circles, respectively. **(F)** Side view from the position of the eye icon in **(E)**.

In the course of purifying Ca_V_1.2/Ca_V_β complexes for structural studies, our lab succeeded in isolating the first example of an ion channel:chaperone complex and channel assembly intermediate [24]. Cryo-electron microscopy studies (cryo-EM) of purified, recombinant human Ca_V_1.2 and rabbit Ca_V_β_3_ identified a large (~0.6 MDa), stable complex in which the two-channel components were bound to a nine protein ER chaperone complex known as the EMC, a chaperone thought to aid the insertion of tail-anchored proteins [25–28] and transmembrane segments having mixed hydrophobic/hydrophilic character, such as those in ion channels [29–31], receptors [32–34], and transporters [30,32,35] (Figure 2A–B). Despite much study and suggestion that the EMC could function as a holdase for partly folded membrane proteins [29,34], there have been no structural examples showing how the EMC might engage a client. Hence, the EMC:Ca_V_1.2/Ca_V_β complex not only provides the first view of an ion channel:chaperone complex but also the first view of an EMC:client complex.

The two channel proteins each have a binding site on the opposite face of the EMC from where membrane insertion activity occurs (Figure 2B). The transmembrane component of the channel, the Ca_V_1.2 Ca_V_α_1_ subunit, binds to a site termed the “TM dock” in which the first Ca_V_1.2 voltage sensor domain (VSD I) binds the EMC1 subunit through a large (~1000 Å^2^) interface that spans the membrane. The TM dock has two elements: an interaction site comprising the EMC1 transmembrane helix and S1 and S2 helices from Ca_V_1.2 VSD I framed by a set of hydrophilic interactions at each membrane boundary (Lumenal site and Inner Leaflet site; Figure 2C) and an interaction between the EMC1 brace/crossbar helix that lies on the lumenal side of the bilayer and the Ca_V_1.2 VSD I/PD II interface. The cytoplasmic Ca_V_β subunit binds to a site on the intracellular bulb of the EMC termed the “Cyto dock”. This protein–protein interaction has a similar sized footprint to the TM dock (~1000 Å^2^) and comprises two elements: a smaller site where the Ca_V_β_3_ α5-α6 helices contact EMC2 and a larger site where the Ca_V_β_3_ α3-α4 loop interacts with EMC8 (Figure 2D). Notably, the interactions made by Ca_V_1.2 and Ca_V_β are conserved throughout the seven Ca_V_1 and Ca_V_2 and four Ca_V_β isoforms [24]. Biochemical purification and mass spectrometry show that in the absence of Ca_V_β_3_, Ca_V_1.2 binds the EMC, and that Ca_V_β _3_ does not bind the EMC on its own [24], indicating that Ca_V_β is a critical element for stabilizing the EMC:Ca_V_α_1_/Ca_V_β complex. Importantly, mutations that disrupt the TM and Cyto dock sites reduce channel cell surface expression [24]. Further, the most disruptive Ca_V_β_3_ mutations cause a loss of EMC binding as measured by quantitative mass spectrometry [24]. Together, this functional and biochemical evidence indicates that the EMC:Ca_V_1.2/Ca_V_β_3_ complex is a productive step in Ca_V_ assembly.

Expression of all three channel subunits Ca_V_1.2, Ca_V_β_3_, and Ca_V_α_2_δ-1 yielded purified samples in which the EMC: Ca_V_1.2/Ca_V_β_3_ complex and fully assembled Ca_V_1.2/Ca_V_β_3_/Ca_V_α_2_δ-1 channels were found in similar proportions [24], enabling the first structural determination of Ca_V_1.2 [24]. Comparison of the structures of the EMC-bound state with the assembled Ca_V_1.2 channel [24] revealed two key points: (1) interaction with the EMC drives a set of rigid body movements that affect nearly every part of the Ca_V_1.2/Ca_V_β_3_ complex (Figure 2E–F), and (2) the EMC and Ca_V_α_2_δ bind to the same Ca_V_α_1_ face in a mutually exclusive manner. Notably, key changes in the EMC-bound channel occur in the three elements that form the Ca_V_α_2_δ contact points: the external part of VSD I and the PD II and PD III extracellular loops. Binding of Ca_V_β to the EMC Cyto dock drives dramatic conformational changes in Ca_V_α_1_ that involve a rotation of Ca_V_β away from the membrane plane (Figure 2F). This change orchestrates a coordinated displacement of the Ca_V_1.2 α-interaction domain (AID) that forms the high-affinity binding site for all Ca_V_β isoforms [37–39], VSD II, and PD III. The AID/Ca_V_β_3_ unit tilts ~15° away from the membrane plane, displacing the AID C-terminal end by~11 Å relative to its Ca_V_1.2/Ca_V_β_3_/Ca_V_α_2_δ-1 position (Figure 2F) and enabling four AID acidic residues (D439, E445, D446, and D448) to make a new set of interactions with five basic residues (R507, R511, R514, R515, and R518) from the VSD II IIS0 helix. This AID segment is disordered in Ca_V_1.2/Ca_V_β_3_/Ca_V_α_2_δ-1 and other assembled Ca_V_ structures [40–48] and the interaction forms because IIS0 becomes more helical in the EMC complex. Hence, it appears that these electrostatic interactions provide a link that pulls VSD II and PD III away from the channel central axis as a consequence of the AID tilt, allowing the VSD III/VSD II/AID/Ca_V_β assembly to act as a unit. The most striking outcome of these movements is the partial extraction of PD III from the core of the Ca_V_1.2 pore. This change widens the pore diameter by~3 Å [24], renders PD III unable to make its native quaternary contacts with the other PDs, and leads to the destabilization of VSD III (Figure 2E). Remarkably, the PD III tertiary structure is preserved, underscoring the ability of PDs to fold autonomously [5]. Notably, the VSD III loop that interacts with Ca_V_α_2_δ is unfolded. In addition to these changes, VSD I is rotated ~20° away from its position in the assembled Ca_V_1.2 complex. Overall, these alterations dramatically reshape the channel in a way that splays open the Ca_V_α_2_δ binding site, suggesting that the EMC acts as a holdase for the partially assembled channel and prepares it for handoff to Ca_V_α_2_δ to complete assembly.

### A proposed pathway for Ca_V_ assembly

Based on the ability of the Ca_V_α_1_ subunit to bind the EMC, the strong affinity of the Ca_V_α_1_/Ca_V_β complex for the EMC, structural differences between the EMC-bound and assembled states, and functional consequences of disruption of the EMC:Ca_V_ interface [24], we propose the following working model for Ca_V_ biogenesis and assembly. Following Ca_V_α_1_ synthesis and membrane insertion by pathways that are not well understood and that may involve steps as outlined in (Figure 1C), Ca_V_α_1_ binds to the EMC TM dock site. The stability of this complex is enhanced by the addition of Ca_V_β forming the EMC:Ca_V_α_1_/Ca_V_β holdase complex, a step that could explain the ability of Ca_V_βs to protect Ca_V_α_1_ pore-forming subunits from proteasome and ERAD pathway [49] degradation in a variety of cell types, including neurons [49,50]. It should be noted that it is also possible that pre-formed Ca_V_α_1_/Ca_V_β complexes directly bind the EMC providing a second route to the holdase complex. Designing experiments to probe these early steps of Ca_V_ biogenesis is an important goal for clarifying these pathways.

**Figure 3:**
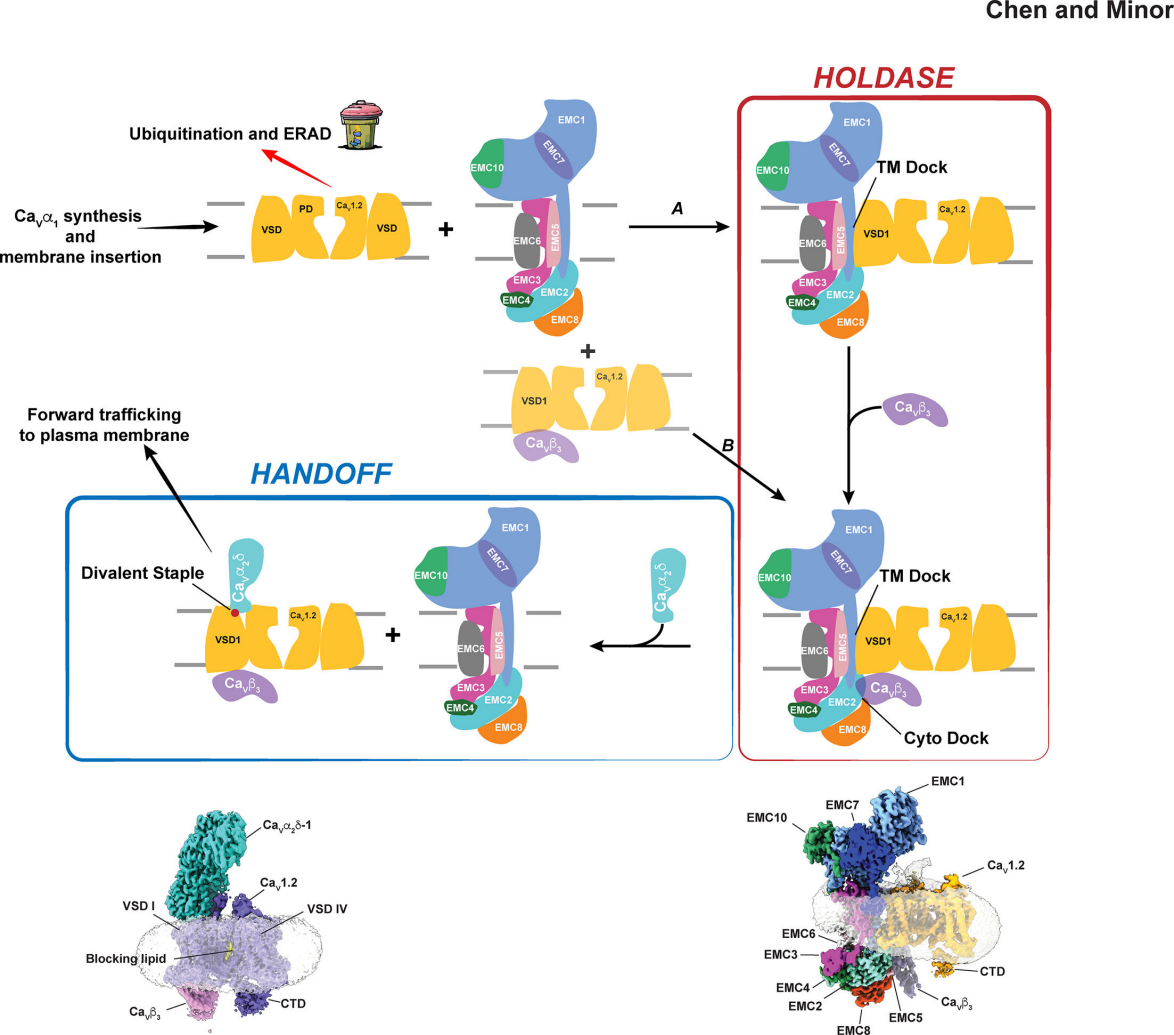
Proposed Ca_V_ assembly pathway. Following Ca_V_α_1_ synthesis and membrane insertion, Ca_V_α_1_s can be tagged for degradation or protected by the formation of the EMC:Ca_V_ holdase complex. Association with the EMC can happen by an ordered pathway. **(A)** where Ca_V_α_1_ binds first, followed by Ca_V_β or by **(B)** direct association of Ca_V_α_1_/Ca_V_β complexes with the EMC. Release from the Holdase complex requires a handoff of the Ca_V_α_1_/Ca_V_β pair from the EMC to Ca_V_α_2_δ. Experimental structures of intermediates [24] are shown. Assembled Ca_V_ includes the “blocking lipid” (yellow) found in the channel pore [24].

Holdase complex formation is followed by a handoff step in which the partially assembled Ca_V_α_1_/Ca_V_β channel is passed to Ca_V_α_2_δ in a process that has two key components: the completion of a divalent ion binding site between Ca_V_α_2_δ and VSD I and ordering of a set of Ca_V_ elements that comprise the Ca_V_α_2_δ binding sites on PD II and PD III [24]. Whether this process occurs in an ordered way or via a “bind and release” mechanism is not known. However, it is interesting to speculate that there could be a concerted way in which the extracted PD III and availability of its extracellular loop, which is important for Ca_V_α_2_δ binding [51] could serve as an initiation point for the handoff. The large PD III extracellular loops are disordered in the EMC:Ca_V_1.2/Ca_V_β_3_ complex but make extensive interactions with Ca_V_α_2_δ, indicating that folding of these elements is Ca_V_α_2_δ-dependent. Movement of PD II and PD III to their native positions would seem to require the release of Ca_V_β from the Cyto dock and disruption of the VSD II:AID interactions that are formed in the EMC complex. Rigid body rotation of VSD I between its EMC-bound and Ca_V_α_2_δ bound positions completes the coordination sphere of the divalent ion shared by the Ca_V_α_2_δ metal ion-dependent adhesion (MIDAS) site and VSD I (a site we term the “divalent staple”) [24] and completes the consolidation of Ca_V_α_2_δ interactions with its three contact points on Ca_V_α_1_. Ca_V_α_2_δ coordination of divalent ions by the MIDAS site is critical for Ca_V_α_2_δ binding to Ca_V_1.2 [52] and Ca_V_2.2 [53] and for the ability of Ca_V_α_2_δ to promote Ca_V_1.2, Ca_V_2.1, and Ca_V_2.2 plasma membrane trafficking [52–54]. This VSD I residue is conserved in Ca_V_1s and Ca_V_2s [24] and its mutation affects Ca_V_α_2_δ-dependent Ca_V_1 [52] and Ca_V_2 [53] trafficking, supporting the idea that the EMC effects on VSD I conformation influence a key maturation step that is thought to occur in the ER lumen [54]. The result of the handoff would be formation of the native Ca_V_α_1_/Ca_V_β/Ca_V_α_2_δ assembly and license of the channel to leave the ER to continue to the plasma membrane [12]. Currently, there is structural data for only two of the many possible intermediates in this proposed pathway (assembled Ca_V_1.2 and the EMC: Ca_V_1.2/Ca_V_β complex) [24] (Figure 3). Hence, it will be important to probe this potential Ca_V_ assembly route to understand if other intermediates are involved, how key steps such as the association of the channel with the EMC and handoff to Ca_V_α_2_δ take place, and whether there are other factors that participate in the assembly of the channel.

### Key open questions

As the first example of an ion channel:chaperone complex and channel assembly intermediate the EMC: Ca_V_ complex naturally leaves more outstanding questions than it answers. Do other Ca_V_s and Ca_V_βs interact with the EMC similarly? Given the strong conservation of the Ca_V_α_1_ and Ca_V_β TM dock and Cyto dock interface residues, this possibility seems likely [24], but experimental validation will be important as it may reveal variations on the overall theme. Understanding the roles of key channel elements previously implicated in trafficking also poses important questions. The Ca_V_1.2 C-terminal tail is implicated in trafficking [55] but both the full-length channel and Ca_V_1.2 truncated just after the calmodulin-binding domain bound to the EMC similarly [24]. Deeper studies of the role of this channel segment as well as calmodulin, which also affects trafficking [56], are warranted to understand if these elements act at the EMC bound or later stage of channel maturation. Do some disease mutations and drugs cause functional effects by interfering with the formation of the EMC complex? Ca_V_α_2_δ is the receptor for widely-used gabapentinoid anti-nociceptive and anti-anxiety drugs [16,57–60] thought to act by affecting the Ca_V_ numbers on the cell surface expression [57,61,62]. This type of mechanism demonstrates the power of controlling channel number to influence excitability and highlights the possibility that agents that perturb the EMC-bound step might offer a similarly potent means to affect the channel function. Developing agents to probe EMC:Ca_V_ interactions will be an important tool for addressing such issues. EMC:Ca_V_ interactions affect channel expression in heterologous cell systems routinely used to assess channel mechanism, mutations, and drug action [24]. Given the ubiquitous expression of the EMC, one would like to know whether EMC: Ca_V_ complexes form in native cells.

How general is the example set by the EMC: Ca_V_ complex? Many VGIC superfamily members share the core VSD architecture that interacts with the TM dock site, raising the possibility that other VGIC classes are EMC-dependent. Defining whether other channels interact with the EMC in ways that are similar to Ca_V_s is a key question. Although Ca_V_β is unique to the Ca_V_ subfamily, it is not difficult to imagine that the large intracellular domains found in many VGICs could interact with the Cyto dock. In this regard, it will be interesting to address whether the Ca_V_3 subfamily [3] that does not depend on either Ca_V_β or Ca_V_α_2_δ is EMC-dependent. Finally, the EMC has a multitude of diverse clients [27]. The EMC:Ca_V_ structure provides the first example of an EMC:client complex. Understanding whether other types of membrane proteins use the TM dock and Cyto dock sites or use other sites, such as the EMC3/4/7 interface found to bind the outer mitochondrial membrane protein VDAC [63], is an important avenue of study.

Do other chaperones act as holdases for channel subunits? The recent report of the structure of a GABA_A_ receptor subunit with a chaperone, NACHO, that acts on a subset of pentameric ligand-gated ion channels shows that this ER-resident transmembrane protein sequesters a key intermembrane interface in the absence of the other subunits required to make the functional channel [64]. This exciting finding has parallels with the EMC:Ca_V_ complex that highlights the role of chaperones in protecting partially assembled states of ion channels and underscores the need to define both general and specific mechanisms by which chaperones aid ion channel assembly.

Assembly from parts is the first step in the life of every ion channel. The door is now open to explore the structural principles that underlie how these marvelous entities at the core of the bioelectric signals that are required for life are put together from their various components. Probing the principles that govern ion channel assembly will not only fill a largely blank space in our knowledge of ion channel structure and function, but will offer new opportunities to understand how channel function goes wrong in disease states and inspire the development of new ways to control channel function.

**Perspectives**

- Understanding how ion channel complexes are assembled is of paramount importance for elucidating their normal function and regulation as well as the effects of disease mutations and drugs.
- The discovery of the role of the EMC in Ca_V_ assembly highlights the role of chaperone proteins in ion channel assembly and provides a framework for probing the structural underpinnings of ion channel biogenesis.
- Defining whether other ion channels are EMC dependent, if other chaperone systems are important for channel biogenesis and assembly, and whether such complexes form in native cells is an important goal.

## Abbreviations

AID: α-interaction domain
Cav: voltage-gated calcium channel
EMC: endoplasmic membrane protein complex
ER: endoplasmic reticulum
MIDAS: metal ion-dependent adhesion site
PD: pore domain
SF: selectivity filter
VGIC: voltage-gated ion channel
VSD: voltage sensor domain

## References

[1] Isacoff E.Y., Jan L.Y., Minor D.L. (2013). Conduits of life’s spark: a perspective on ion channel research since the birth of neuron. Neuron.

[2] Yu F.H., Yarov-Yarovoy V., Gutman G.A., Catterall W.A (2005). Overview of molecular relationships in the voltage-gated ion channel superfamily. Pharmacol. Rev..

[3] Zamponi G.W., Striessnig J., Koschak A., Dolphin A.C., Sibley D.R (2015). The physiology, pathology, and pharmacology of voltage-gated calcium channels and their future therapeutic potential. Pharmacol. Rev..

[4] Li Q., Wanderling S., Paduch M., Medovoy D., Singharoy A., McGreevy R. (2014). Structural mechanism of voltage-dependent gating in an isolated voltage-sensing domain. Nat. Struct. Mol. Biol..

[5] Arrigoni C., Lolicato M., Shaya D., Rohaim A., Findeisen F., Fong L.-K. (2022). Quaternary structure independent folding of voltage-gated ion channel pore domain subunits. Nat. Struct. Mol. Biol..

[6] Deutsch C (2003). The birth of a channel. Neuron.

[7] Boström K., Wettesten M., Borén J., Bondjers G., Wiklund O., Olofsson S.O (1986). Pulse-chase studies of the synthesis and intracellular transport of apolipoprotein B-100 in Hep G2 cells. J. Biol. Chem..

[8] Ingolia N.T., Lareau L.F., Weissman J.S (2011). Ribosome profiling of mouse embryonic stem cells reveals the complexity and dynamics of mammalian proteomes. Cell.

[9] Buraei Z., Yang J (2010). The ß subunit of voltage-gated Ca2+ channels. Physiol. Rev..

[10] Dolphin A.C (2016). Voltage-gated calcium channels and their auxiliary subunits: physiology and pathophysiology and pharmacology. J. Physiol. (Lond.).

[11] Ben-Johny M., Yue D.T (2014). Calmodulin regulation (calmodulation) of voltage-gated calcium channels. J. Gen. Physiol..

[12] Ferron L., Koshti S., Zamponi G.W (2021). The life cycle of voltage-gated Ca^2+^ channels in neurons: an update on the trafficking of neuronal calcium channels. Neuronal Signal.

[13] Singer D., Biel M., Lotan I., Flockerzi V., Hofmann F., Dascal N (1991). The roles of the subunits in the function of the calcium channel. Science.

[14] Shistik E., Ivanina T., Puri T., Hosey M., Dascal N (1995). Ca2+ current enhancement by alpha 2/delta and beta subunits in Xenopus oocytes: contribution of changes in channel gating and alpha 1 protein level. J. Physiol. (Lond.).

[15] Gurnett C.A., De Waard M., Campbell K.P (1996). Dual function of the voltage-dependent Ca2+ channel alpha 2 delta subunit in current stimulation and subunit interaction. Neuron.

[16] Davies A., Hendrich J., Van Minh A.T., Wratten J., Douglas L., Dolphin A.C (2007). Functional biology of the α2δ subunits of voltage-gated calcium channels. Trends Pharmacol. Sci..

[17] Li Q., Wanderling S., Sompornpisut P., Perozo E (2014). Structural basis of lipid-driven conformational transitions in the KvAP voltage-sensing domain. Nat. Struct. Mol. Biol.

[18] Chakrapani S., Sompornpisut P., Intharathep P., Roux B., Perozo E (2010). The activated state of a sodium channel voltage sensor in a membrane environment. Proc. Natl. Acad. Sci. U.S.A.

[19] Taylor K.C., Kang P.W., Hou P., Yang N.-D., Kuenze G., Smith J.A. (2020). Structure and physiological function of the human KCNQ1 channel voltage sensor intermediate state. Elife.

[20] Butterwick J.A., MacKinnon R (2010). Solution structure and phospholipid interactions of the isolated voltage-sensor domain from KvAP. J. Mol. Biol..

[21] Delaney E., Khanna P., Tu L., Robinson J.M., Deutsch C (2014). Determinants of pore folding in potassium channel biogenesis. Proc. Natl. Acad. Sci. U.S.A.

[22] Gajewski C., Dagcan A., Roux B., Deutsch C (2011). Biogenesis of the pore architecture of a voltage-gated potassium channel. Proc. Natl. Acad. Sci. U.S.A.

[23] McDonald S.K., Levitz T.S., Valiyaveetil F.I (2019). A shared mechanism for the folding of voltage-gated k(+) channels. Biochemistry.

[24] Chen Z., Mondal A., Abderemane-Ali F., Jang S., Niranjan S., Montaño J.L. (2023). EMC chaperone-Ca_V_ structure reveals an ion channel assembly intermediate. Nature.

[25] Jonikas M.C., Collins S.R., Denic V., Oh E., Quan E.M., Schmid V. (2009). Comprehensive characterization of genes required for protein folding in the endoplasmic reticulum. Science.

[26] Christianson J.C., Olzmann J.A., Shaler T.A., Sowa M.E., Bennett E.J., Richter C.M. (2011). Defining human ERAD networks through an integrative mapping strategy. Nat. Cell Biol..

[27] Hegde R.S (2022). The function, structure, and origins of the ER membrane protein complex. Annu. Rev. Biochem..

[28] Guna A., Volkmar N., Christianson J.C., Hegde R.S (2018). The ER membrane protein complex is a transmembrane domain insertase. Science.

[29] Richard M., Boulin T., Robert V.J.P., Richmond J.E., Bessereau J.-L (2013). Biosynthesis of ionotropic acetylcholine receptors requires the evolutionarily conserved ER membrane complex. Proc. Natl. Acad. Sci. U.S.A.

[30] Talbot B.E., Vandorpe D.H., Stotter B.R., Alper S.L., Schlondorff J.S (2019). Transmembrane insertases and N-glycosylation critically determine synthesis, trafficking, and activity of the nonselective cation channel TRPC6. Journal of Biological Chemistry.

[31] Coelho J.P.L., Stahl M., Bloemeke N., Meighen-Berger K., Alvira C.P., Zhang Z.-R (2019). A network of chaperones prevents and detects failures in membrane protein lipid bilayer integration. Nat. Commun.

[32] Satoh T., Ohba A., Liu Z., Inagaki T., Satoh A.K (2015). dPob/EMC is essential for biosynthesis of rhodopsin and other multi-pass membrane proteins in Drosophila photoreceptors. Elife.

[33] Chitwood P.J., Juszkiewicz S., Guna A., Shao S., Hegde R.S (2018). EMC is required to initiate accurate membrane protein topogenesis. Cell.

[34] Miller-Vedam L.E., Bräuning B., Popova K.D., Schirle Oakdale N.T., Bonnar J.L., Prabu J.R. (2020). Structural and mechanistic basis of the EMC-dependent biogenesis of distinct transmembrane clients. Elife.

[35] Shurtleff M.J., Itzhak D.N., Hussmann J.A., Schirle Oakdale N.T., Costa E.A., Jonikas M. (2018). The ER membrane protein complex interacts cotranslationally to enable biogenesis of multipass membrane proteins. Elife.

[36] Hegde R.S., Keenan R.J (2024). A unifying model for membrane protein biogenesis. Nat. Struct. Mol. Biol..

[37] Van Petegem F., Duderstadt K.E., Clark K.A., Wang M., Minor D.L. (2008). Alanine-scanning mutagenesis defines a conserved energetic hotspot in the CaValpha1 AID-CaVbeta interaction site that is critical for channel modulation. Structure.

[38] Van Petegem F., Clark K.A., Chatelain F.C., Minor D.L. (2004). Structure of a complex between a voltage-gated calcium channel beta-subunit and an alpha-subunit domain. Nature.

[39] Pragnell M., De Waard M., Mori Y., Tanabe T., Snutch T.P., Campbell K.P (1994). Calcium channel β-subunit binds to a conserved motif in the I–II cytoplasmic linker of the α1-subunit. Nature.

[40] Yao X., Wang Y., Wang Z., Fan X., Wu D., Huang J. (2022). Structures of the R-type human Ca_v_2.3 channel reveal conformational crosstalk of the intracellular segments. Nat. Commun..

[41] Yao X., Gao S., Yan N (2022). Structural basis for pore blockade of human voltage-gated calcium channel Ca_v_1.3 by motion sickness drug cinnarizine. Cell Res..

[42] Yao X., Gao S., Wang J., Li Z., Huang J., Wang Y (2022). Structural basis for the severe adverse interaction of sofosbuvir and amiodarone on L-type Ca_v_ channels. Cell.

[43] He L., Yu Z., Geng Z., Huang Z., Zhang C., Dong Y (2022). Structure, gating, and pharmacology of human CaV3.3 channel. Nat. Commun..

[44] Gao S., Yao X., Yan N (2021). Structure of human Cav2.2 channel blocked by the painkiller ziconotide. Nature.

[45] Zhao Y., Huang G., Wu Q., Wu K., Li R., Lei J (2019). Cryo-EM structures of apo and antagonist-bound human Cav3.1. Nature.

[46] Zhao Y., Huang G., Wu J., Wu Q., Gao S., Yan Z (2019). Molecular basis for ligand modulation of a mammalian voltage-gated Ca^2+^ channel. Cell.

[47] Wu J., Yan Z., Li Z., Qian X., Lu S., Dong M. (2016). Structure of the voltage-gated calcium channel Ca(v)1.1 at 3.6 Å resolution. Nature.

[48] Wu J., Yan Z., Li Z., Yan C., Lu S., Dong M. (2015). Structure of the voltage-gated calcium channel Cav1.1 complex. Science.

[49] Altier C., Garcia-Caballero A., Simms B., You H., Chen L., Walcher J. (2011). The Cavβ subunit prevents RFP2-mediated ubiquitination and proteasomal degradation of L-type channels. Nat. Neurosci..

[50] Waithe D., Ferron L., Page K.M., Chaggar K., Dolphin A.C (2011). Beta-subunits promote the expression of Ca(V)2.2 channels by reducing their proteasomal degradation. J. Biol. Chem..

[51] Gurnett C.A., Felix R., Campbell K.P (1997). Extracellular interaction of the voltage-dependent Ca2+ channel α2δ and α1 Subunits. J. Biol. Chem..

[52] Bourdin B., Briot J., Tétreault M.-P., Sauvé R., Parent L (2017). Negatively charged residues in the first extracellular loop of the L-type CaV1.2 channel anchor the interaction with the CaVα2δ1 auxiliary subunit. Journal of Biological Chemistry.

[53] Dahimene S., Page K.M., Kadurin I., Ferron L., Ho D.Y., Powell G.T. (2018). The α2δ-like protein Cachd1 increases N-type calcium currents and cell surface expression and competes with α2δ-1. Cell Rep..

[54] Cantí C., Nieto-Rostro M., Foucault I., Heblich F., Wratten J., Richards M.W. (2005). The metal-ion-dependent adhesion site in the Von Willebrand factor-A domain of alpha2delta subunits is key to trafficking voltage-gated Ca2+ channels. Proc. Natl. Acad. Sci. U.S.A..

[55] Fang K., Colecraft H.M (2011). Mechanism of auxiliary beta-subunit-mediated membrane targeting of L-type (Ca(V)1.2) channels. J Physiol.

[56] Wang H.G., George M.S., Kim J., Wang C., Pitt G.S (2007). Ca2+/calmodulin regulates trafficking of Ca(V)1.2 Ca2+ channels in cultured hippocampal neurons. J. Neurosci.

[57] Field M.J., Cox P.J., Stott E., Melrose H., Offord J., Su T.-Z. (2006). Identification of the alpha2-delta-1 subunit of voltage-dependent calcium channels as a molecular target for pain mediating the analgesic actions of pregabalin. Proc. Natl. Acad. Sci. U.S.A..

[58] Dolphin A.C (2018). Voltage-gated calcium channel α2δ subunits: an assessment of proposed novel roles. F1000Res..

[59] Chen Z., Jr D.L (2023). Structural basis for Ca(V)alpha(2)delta:gabapentin binding. Nat. Struct. Mol. Biol.

[60] Kozai D., Numoto N., Nishikawa K., Kamegawa A., Kawasaki S., Hiroaki Y (2023). Recognition mechanism of a novel gabapentinoid drug, mirogabalin, for recombinant human α2δ1, a voltage-gated calcium channel subunit. J. Mol. Biol.

[61] Bauer C.S., Nieto-Rostro M., Rahman W., Tran-Van-Minh A., Ferron L., Douglas L. (2009). The increased trafficking of the calcium channel subunit alpha2delta-1 to presynaptic terminals in neuropathic pain is inhibited by the alpha2delta ligand pregabalin. J. Neurosci..

[62] Cassidy J.S., Ferron L., Kadurin I., Pratt W.S., Dolphin A.C (2014). Functional exofacially tagged N-type calcium channels elucidate the interaction with auxiliary α _2_ δ-1 subunits. Proc. Natl. Acad. Sci. U.S.A.

[63] Li M., Zhang C., Xu Y., Li S., Huang C., Wu J. (2024). Structural insights into human EMC and its interaction with VDAC. Aging (Milano).

[64] Hooda Y., Sente A., Judy R.M., Smalinskaitė L., Chew S.-Y.P., Naydenova K. (2024). Mechanism of NACHO-mediated assembly of pentameric ligand-gated ion channels. Biochemistry.

